# Integrated Management of Diabetes and Tuberculosis in Rural India – Results From a Pilot Study

**DOI:** 10.3389/fpubh.2022.766847

**Published:** 2022-05-10

**Authors:** Rohina Joshi, Deepanjali Behera, Gian Luca Di Tanna, Mohammed Abdul Ameer, Kenneth Yakubu, Devarsetty Praveen

**Affiliations:** ^1^School of Population Health, Faculty of Medicine, University of New South Wales, Sydney, NSW, Australia; ^2^The George Institute for Global Health, Faculty of Medicine, University of New South Wales, Sydney, NSW, Australia; ^3^Health Systems Science, The George Institute for Global Health, New Delhi, India; ^4^KIIT School of Public Health, KIIT University, Bhubaneswar, India; ^5^Prasanna School of Public Health, Manipal Academy of Higher Education, Manipal, India

**Keywords:** delivery of health care (MeSH), diabetes mellitus, tuberculosis, primary health care (MeSH), feasibility studies

## Abstract

**Introduction:**

The World Health Organization and International Union against Tuberculosis (TB) recommends screening patients with TB for Diabetes Mellitus (DM) at the initiation of treatment. There are few pilot studies which screen TB patients for DM, but none of them have documented the feasibility of managing TB patients with DM in the Indian healthcare setting. Operational research is needed to determine the best way to manage individuals with both conditions. This pilot study aimed to develop, and field test an integrated, multidisciplinary program addressing the management of individuals with TB and DM and other associated chronic conditions in the Indian primary healthcare setting.

**Methods:**

This pilot study used a randomized controlled trial design with mixed-methods evaluation and was conducted in Guntur district of Andhra Pradesh, a southern state of India. All the 120 patients newly diagnosed with TB from 10 participating villages were screened for DM and associated cardiovascular risk factors. Non-physician health workers were trained to follow-up patients for a period of 8 months to encourage treatment adherence, monitor treatment response including blood glucose levels and provide lifestyle advice.

**Results:**

The intervention was well-accepted by the providers and patients. However, there were no statistically significant variations observed for mean blood glucose levels (mean [SD]: 5.3 [−23.3 to 33.8]) of patients for both intervention and control group participants in this feasibility study. Awareness about diabetes and tuberculosis comorbidity and cardiovascular risk increased among the non-physician health workers in the intervention arm of the study.

**Discussion:**

The co-management of TB-DM is acceptable to both the health providers and patients. With appropriate training, availability of infrastructure and planned intervention implementation, it is feasible to co-manage TB-DM within the existing primary health care system in India.

## Introduction

There is strong evidence about the epidemiological linkage between tuberculosis (TB) and type 2 diabetes mellitus (DM) (1). Globally, 15% of adult TB cases are estimated to be attributable to DM which is nearly the same for HIV-TB association (2). Having DM increases the risk of getting infected with TB infection by 3-fold (1, 3, 4). Coexistence of TB-DM also interferes with treatment outcomes and exacerbate complications (1). Studies have reported that patient with TB and DM have delayed sputum smear conversion, higher chances of TB relapse, treatment failure and death as compared to those without DM (4–6). Conversely, TB also affects glycemic control leading to hyperglycemia among patients (7, 8).

India has the highest burden of TB-DM comorbidity worldwide (4). In 2017, 27% of estimated global TB patients were living in India, with about 410,000 TB related deaths among non-HIV infected population (9). Simultaneously, a fast-emerging economy, demographic and epidemiological transition, and rapid urbanization have resulted in a dramatic increase in DM prevalence in the country. The International Diabetes Federation estimated that 72.9 million Indians had Dm in 2017 (10) and number is projected to increase to 101.2 million in 2030 (10). The anticipated escalating trend of DM epidemic might impede the achievement of End TB strategy by 2025 (11).

To address this emerging challenge in many countries, the World Health Organization (WHO) and the International Union developed a collaborative framework in 2011 which suggested bidirectional screening and combined approaches of care and control for DM and TB (12). Subsequently, the Indian government mandated bidirectional screening of patients with TB and DM (13). Few studies have reported the feasibility of DM screening among patients with TB (14–16), but none of them have documented combined management within Indian health care setting.

The population based non-communicable disease screening program in India currently requires a basic cardiovascular disease (CVD) risk assessment at the community level by the Accredited Social Health Activists (ASHAs), who are India's community health workers, followed by referral and screening of BP, blood sugar (BS) and some of the common cancers for population above 30 years of age by the auxiliary nurse midwife (ANMs) at the sub-center level (17). The patient is then referred to the closest primary health center for diagnoses and treatment (17). There are also clear guidelines for the management of TB and access to free services under the revised national TB control (RNTCP) program (11). However, the integrated care for patients with TB and DM remains a distant dream. Despite India's national framework for joint TB-DM collaborative activities implemented since 2017, the follow-up and joint intervention activities for TB-DM comorbid patients remains ambiguous (13). Also, the population-based screening process for common NCDs under the National Program for the Prevention and control of Cancer, Diabetes, Cardiovascular Disease and Stroke (NPCDCS) program is recommended to be followed for patients with hypertension and DM annually (18), while patients with TB-DM comorbidity need more frequent screening and follow-up. Hence, an integrated intervention protocol on community-based follow-up and care for patients with TB-DM in India is essential for better management and preventing serious consequences of this deadly comorbidity.

In this pilot study, we assessed the feasibility of integrating the screening and management of DM and related chronic conditions such as CVD within the existing TB program of India. It incorporated key strategies of task-sharing between the primary care physician and the ANM (non-physician health worker) and use of technology in providing evidence-based health care. The aim of the study was to develop and evaluate the feasibility, acceptability, and preliminary effectiveness of a complex intervention for the integrated screening and management of DM and TB at the community level.

## Materials and Methods

### Design, Setting and Randomization

This pilot study used a cluster randomized controlled trial design with mixed-methods evaluation and was conducted in Guntur district of Andhra Pradesh, a southern state of India. Recruitment took place from May-October 2017 with an 8-month follow-up period. The end of study evaluation took place from Jan-June 2018. We invited 10 primary health care centers (PHCs) with co-located functional Directly Observed Treatment Short-term (DOTS) to participate in the study. These PHCs were selected based on the discussion with the district health administration and their proximity to The George Institute's Field Office in the district. Patients meeting the eligibility criteria of being aged 18 years or more and newly diagnosed with TB (defined as diagnosed within the last 4 months) were invited to participate. All ANMs working and reporting to the DOTS centers were also invited to be included in the study. Since this was a pilot study to understand the feasibility and acceptability of the intervention, formal sample size calculation was not carried out. Randomization of all 10 sites was conducted prior to initiation of the intervention. Central computer-based blinded randomisation was conducted and the selected PHCs were allocated to intervention and control arms with five PHCs in each group.

### Development and Training of Intervention Tools

SMARThealth application has been developed by the George Institute for Global Health as an electronic decision support system to facilitate guidelines-based assessment and management of cardiovascular disease risk to be used by lay health workers and doctors (19, 20). The SMARThealth platform was adapted to include screening and management protocols for DM and TB based on Indian and international guidelines (21–23), and was validated by domain experts and physicians. The development of the platform involved a process of summarizing the clinical guidelines, converting this into a programmable algorithm, performing clinical validation of the algorithm and developing a user interface. Details for development and validation of the SMARThealth platform are explained elsewhere (20). The SMARThealth intervention for this study was designed to be used *via* android-based tablets. The ANMs used the tablets to screen patients and receive decision support about referral, management, and follow-up.

Prior to the beginning of intervention, all ANMs working at PHCs of that arm were trained for 2 days on TB, DM, and CVD risk and the use of SMARThealth. The training modules included information on co-morbid disease conditions, use of tablet device to administer the screening tools, interpret the decision support output, refer individuals identified to have DM or CV risk to the PHC; and monitor and promote adherence to any prescribed medications and provide lifestyle advice in these individuals.

### Intervention

At the beginning of the pilot study, all patients diagnosed with TB were enlisted and invited to participate in the study. Demographic and anthropometric information was collected from the consented patients along with their blood pressure measurement. This information was used for identification of individuals with high risk of DM and CVD by the ANMs using the SMARThealth decision support tool. As per the national guidelines, the random blood sugar (RBS) values were used to screen individuals for DM. Patients with ≥110 mg/dl blood glucose levels were referred to the PHC for confirmation of diagnosis. Those with fasting blood sugar (FBG) levels of ≥126 mg/dl were diagnosed to have DM and were referred to the PHC physicians for treatment. ANMs followed up the patients during home visits to enquire about blood glucose lowering treatment initiation, provided medication adherence support, and monitored blood glucose levels during regular DOTS visit in the community. They also educated the individuals and families about the risk factors and advised them about tobacco use, diet, and physical activity. ANMs followed up the patients monthly during the first 3 months, after which they visited them once in 3 months and a final visit after the cessation of the DOTS program (usually coinciding with 8 months post recruitment).

### Control

PHCs and participants in the usual care villages were not offered any component of the intervention. The patients continued to receive usual care from their DOTS providers.

### Data Collection

Data collection was carried out at baseline and at the end of 8 months by trained research associates who conducted independent interviews, collected anthropometry and blood pressure measurements, and tested random blood sugar levels of the study participants. These data were collected electronically using tablets. In addition, in-depth interviews were carried out with randomly selected patients (six in total, one from each intervention village and one additional participant) and ANMs (five in total, one from each intervention village) to understand the acceptability and patient and provider experience of the intervention and explore the barriers and enablers of the integrated disease management approach. Interviews were conducted by research assistants who used interview guides to facilitate the interviews. Interviews were conducted in Telugu. Qualitative data were audio-recorded which were later transcribed and translated to English. Feasibility was defined as the ease of adoption of the intervention while acceptability was defined as the uptake of the intervention by both ANMs (use of SMARThealth) and individuals with DB and TB (if individuals agreed to share their information and allow the ANM to use SMARThealth for the management of their condition).

### Outcomes

The primary outcome of this mixed-method evaluation of the pilot study was to a) define the prevalence of DM in patients with TB (18), b) increase the proportion of individuals with TB screened for DM and associated CVD risk factors by ANMs, c) increase proportion of individuals with DM/TB/high CVD risk on recommended therapies in the intervention arm, d) improved awareness and knowledge among patients about DM and its complications in the intervention arm.

Effectiveness of study intervention were measured in terms of changes in blood glucose, blood pressure, body mass index (BMI) levels, quality of life of patients as well as their risk levels and medication adherence in intervention group as compared to the control group. The feasibility and acceptability of the intervention within the context of routine DOTS program were explored from the user and provider experience information collected through qualitative interviews.

### Analysis

Outcome analysis were done at the individual participant level. Descriptive statistics were conducted for sociodemographic characteristics and clinical parameters for intervention and control group. Differences in outcomes between the Intervention and Control arm have been assessed by linear mixed effects models estimated by restricted maximum likelihood with the clusters (PHC) as random effects. Uni- and multi-variable analyses have been performed with a covariate set defined a priori that included age, gender, education, occupation, BMI, smoking and chewing. Statistical analyses have been performed using Stata v16.

All qualitative data were manually analyzed using thematic content analysis method (24). We used an inductive approach to search for patterns from the transcripts. This allowed for themes to develop during the coding process. Two coders (RJ and DB) reviewed the transcripts and discussed the emerging themes with the senior author (DP). We explored two themes relating to the provider and patient experience. The knowledge, training, acceptability and feasibility of using SMARThealth application by the ANMs for screening and follow-up care for DM and other related chronic diseases at the community level were assessed. We also explored for themes relating to barriers in uptake of the intervention.

### Ethics Approval

Ethical approval was granted by the Independent Ethics Review Committee of Centre for Chronic Disease Control, New Delhi, India. The study was endorsed by Government health officials at the District level in Guntur. Written informed consent was obtained in the local language from all participants prior to randomization.

## Results

**Figure 1:**
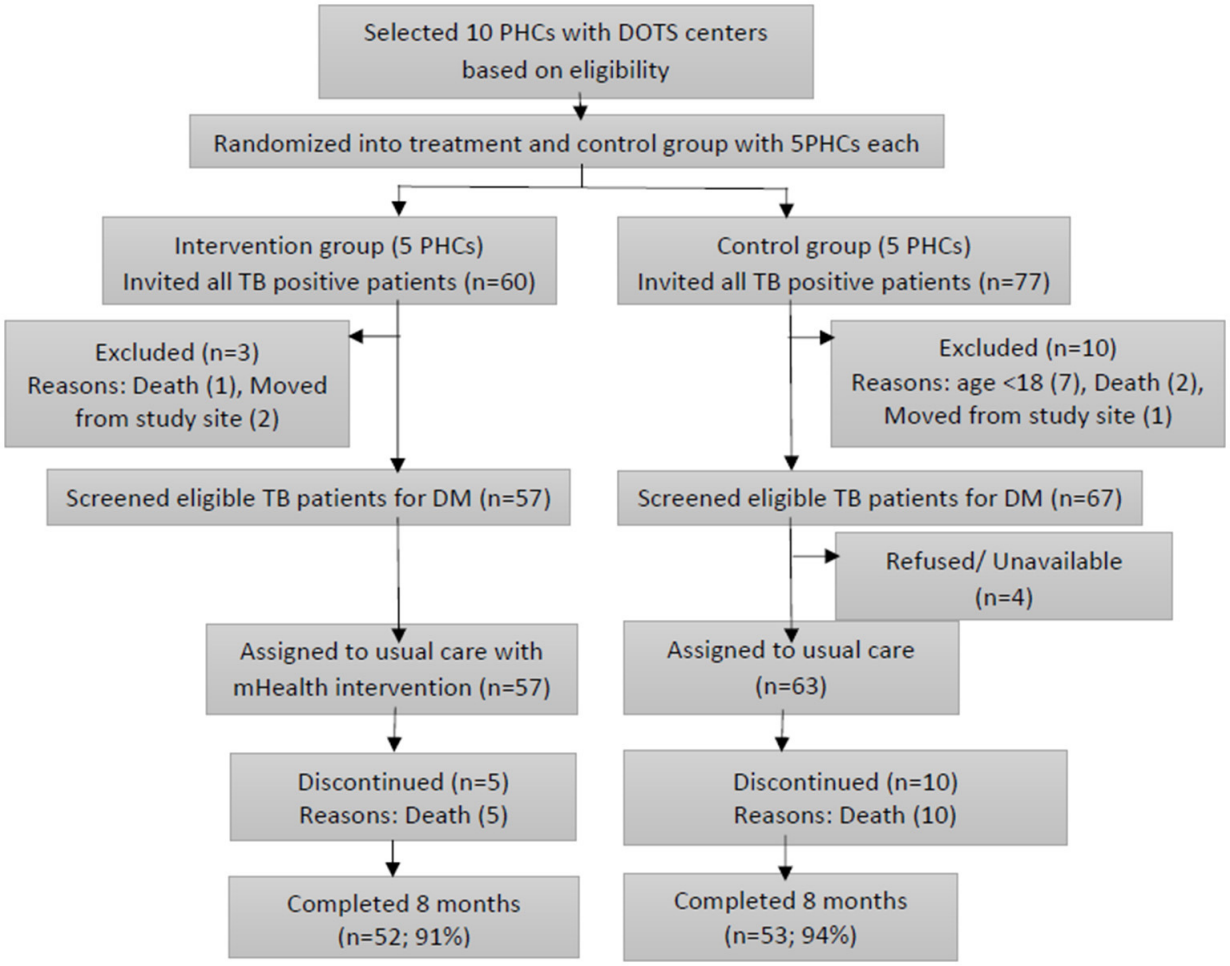
Flowchart of study participants.

Figure 1 depicts the flow chart of randomization and selection of study participants. Ten PHCs participated in the pilot study of which five were randomized to the intervention and five to usual care. All eligible patients (120) gave written informed consent to participate in the study of which 57 were assigned to intervention group and 63 to control group. Thirteen patients were loss to follow up (3 intervention and 10 control arm).

### Sample Characteristics

Table 1 provides the baseline characteristics of study participants. The mean age of participants was 44 years (SD = 13.7) in intervention group and 43 years (SD = 14.7) in control group. The age of the participants ranged from 19 to 75 years. About 70% of the study participants were males and the primary occupation was working as laborers in skilled (29.2%), manual (15.8%) or agricultural (33.3%) activities. About 47.4% participants had no education in intervention group as compared to 27% in control group.

**Table 1:** Baseline characteristics of study respondents in intervention and control group.

**Characteristics**	**Intervention** **(*n* = 57)**	**Control** **(*n* = 63)**	**Total** **(*n* = 120)**
Age (years)	Mean: 44.0, SD: 13.7	Mean: 42.9, SD: 14.7	Mean: 43.4, SD: 14.2
Male	40 (70.2)	42 (66.7)	82 (68.3)
Education			
Illiterate	27 (47.4)	17 (27.0)	44 (36.7)
Class 1–9	21 (36.8)	25 (39.7)	46 (38.3)
Class 10 or above	9 (15.8)	21 (33.3)	30 (25.0)
Occupation			
Agricultural laborer	19 (33.3)	21 (33.3)	40 (33.3)
Manual laborer	8 (14.0)	11 (17.5)	19 (15.8)
Skilled laborer	18 (31.6)	17 (27.0)	35 (29.2)
Business	8 (14.0)	7 (11.1)	15 (12.5)
Others	4 (7.0)	7 (11.1)	11 (9.2)
Current Smoker	15 (48.4)	18 (52.9)	33 (50.8)
Current tobacco chewer	8 (61.5)	4 (44.4)	12 (54.6)
Body mass index^#^	Mean: 20.9, SD: 5.7	Mean: 20.9, SD: 7.1	Mean: 20.9, SD: 6.4
Pre-diagnosed diabetics	11 (19.3)	8 (12.7)	19 (15.8)
CVD risk level			
Level 1	44 (77.2)	50 (79.4)	94 (78.3)
Level 2	8 (14.0)	7 (11.1)	15 (12.5)
Level 3 or above	5 (8.8)	6 (9.5)	11 (9.2)
Systolic blood pressure (mmHg)	Mean: 104.7, SD: 24.1	Mean: 100.9, SD: 20.7	Mean: 102.7, SD: 22.4
Diastolic blood pressure (mmHg)	Mean: 73.5, SD: 15.0	Mean: 70.8, SD: 13.8	Mean: 72.1, SD: 14.4
HIV positive	2 (3.5)	3 (4.8)	5 (4.2)
Quality of life^##^			
Very low	4 (7.0)	3 (4.8)	7 (5.8)
Low	17 (29.8)	20 (31.7)	37 (30.8)
Good	36 (63.2)	37 (58.7)	73 (60.8)

*Values are mentioned as numbers (percentages) unless otherwise stated*.

# *2 missing cases (n = 62 for control and n = 56 for intervention), Calculated as Weight in kilograms divided by square of height in meters*.

## *3 missing cases in control (n = 60), Calculated using WHO (Five) Well-Being Index and categorized as ≤ 28 as very low, 29-50 as low and >50 as good on a scale of 0–100*.

### Prevalence of DM, CVD Risk, and Other Health Indicators

A total of 19 patients with TB (15.8%) had previously been diagnosed to have DM (19.3% in intervention and 12.7% in control group). No new participants were screen-positive for DM during the study. Table 1 outlines the baseline characteristics of the study participants. The mean systolic and mean diastolic blood pressure among the study participants were 102.7 mmHg (SD = 22.4) and 72.1 mmHg (SD = 14.4), respectively. Smoking (27.5%) was more common than chewing tobacco (10%). Five patients reported to have HIV (4.2%).

Table 2 shows the comparison of clinical outcome indicators and their variations from baseline to end-of-study for both intervention and control groups. There were no statistically significant variations observed for mean blood glucose levels of patients from the beginning to the end of study for both intervention and control group participants. For systolic blood pressure, there was a significant increase from baseline to end of study in both the intervention (*p*-value = 0.030) and control groups (*p*-value = 0.001). But the difference in changes across both groups was found to be not statistically significant (difference = −1.0, 95% CI: −7.5 to 5.5, *p*-value = 0.758). Similarly, the variations between intervention and control groups in terms of changes in BMI (−0.1, 95% CI:−0.9 to 0.7, *p*-value = 0.778).

**Table 2:** Outcome variables at baseline and end-of-study and differences between intervention and control group at the end of intervention.

**Measures**	**Intervention**		**Control**				
	**Baseline**	**End-of-study**	**Baseline**	**End-of-study**	**Difference^+^ (95% CI)**	***P* value**	Adj *P* value^*^
	***n* (SD)**	**(SD)**	***n* (SD)**	**(SD)**			
Blood glucose (mg/dl)	139.4 (75.5)	152.3 (89.0)	145.4 (74.2)	151.4 (88.9)	5.3 (-23.3 to 33.8)	0.718	0.467
Systolic blood pressure (mmHg)	104.7 (24.1)	113.4 (21.9)	100.9 (20.7)	107.2 (18.9)	−1.0 (-7.5 to 5.5)	0.758	0.790
Diastolic blood pressure (mmHg)	73.5 (15.0)	77.6 (14.4)	70.8 (13.8)	74.5 (13.7)	−0.3 (-5.2 to 4.5)	0.897	0.674
Body mass index^†^	20.9 (5.7)	22.4 (4.5)	20.9 (7.1)	22.2 (4.9)	−0.1 (-0.9 to 0.7)	0.778	0.536

*Values are mentioned as mean (SD) unless otherwise stated*.

+ *Mean difference between Baseline to end of study changes comparing Intervention and Control group*.

† *2 missing cases for BMI in Baseline (n = 56 in intervention and n = 62 in control) and 2 missing cases in End line (n = 50 in intervention and n = 53 in control)*.

* *p-value from the fully adjusted (multivariable) model*.

### Changes in Risk Factors and Medication Use

The comparison of proportions of risk factors across intervention and control group is provided in Table 3. There was decrease in smoking and tobacco chewing rates from baseline to end-of-study in both intervention and control groups. However, the changes were not statistically significant (*p*-value > 0.05). Similarly, there was no significant change observed for CVD risk levels over the study period. Table 4 gives the proportion of patients using necessary medications according to their health condition. Among patients with hypertension, the proportion of people using blood pressure controlling medications increased from 60% to 80% in intervention group. However, the proportion of patients with DM taking glucose lowering medication decreased from 100% to about 91%. At the end-of-the study, all the participants had completed the DOTs course for TB. About 54% patients in the intervention group and 60% in control group were on intensive phase of TB medication at the beginning of study. At the end of 8 months, all had completed the TB treatment.

**Table 3:** Risk factors at baseline and end-of-study.

**Measures**	**Intervention**		**Control**	
	**Baseline** ***n* = 57**	**End-of-study** ***n* = 52**	**Baseline** ***n* = 63**	**End-of-study** ***n* = 53**
Current smoking as number (%)	15 (48.4)	13 (65.0)	18 (52.9)	9 (39.1)
Current tobacco chewing as number (%)	8 (61.5)	2 (40.0)	4 (44.4)	2 (40.0)
CVD risk level				
Level 1	44 (77.2)	43 (82.7)	50 (79.4)	44 (83.0)
Level 2	8 (14.0)	3 (5.8)	7 (11.1)	4 (7.5)
Level 3 or above	5 (8.8)	6 (11.5)	6 (9.5)	5 (9.4)

*Values are mentioned as number (%)*.

**Table 4:** Medication use at baseline and end-of-study.

**Medication use**	**Intervention**		**Control**	
	**Baseline**	**End-of-study**	**Baseline**	**End-of-study**
Blood pressure medications	3 (5.3)	4 (7.7)	1 (1.6)	3 (5.7)
Lipid lowering medications	1 (1.8)	0 (0.0)	0 (0.0)	0 (0.0)
Anti-platelet therapy	1 (1.8)	0 (0.0)	0 (0.0)	0 (0.0)
Glucose lowering medications	11 (19.3)	10 (19.2)	8 (12.7)	6 (11.3)
Tuberculosis medications				
Intensive phase	31 (54.4)	0 (0.0)	37 (58.7)	0 (0.0)
Continuation phase	26 (45.6)	0 (0.0)	25 (39.7)	0 (0.0)
Anti-retroviral therapy	2 (3.5)	0 (0.0)	3 (4.8)	2 (3.8)

*Values are mentioned as number (%)*.

### Provider Experiences

This intervention was acceptable to the ANMs as it increased their knowledge and awareness about the comorbidity. Awareness about DM and TB comorbidity and cardiovascular risk increased among the ANMs in the intervention arm of the study. They felt knowledgeable and empowered to educate people about the risk of developing cardiovascular disease and help them adopt preventive measures.

“*After this ICDM program has begun, we got to know that TB patient can get diabetes, earlier we didn't know about this association*.” – ANM 1

Most of the ANMs thought the training was adequate for them to complete the assigned tasks. However, some felt the need for longer practical training on use of the application, uploading and saving data.

“*Two days training is not sufficient is what I feel …. another whole day should be for training regarding how to upload…. that way we can learn completely” – ANM 2*

At times, ANMs required re-training on the job and contacted the Field Supervisor for additional support.

“*If there was any problem, we called the person who had trained us, they've given us instructions and we have followed the same.” - ANM 5*

As the intervention was integrated into their routine work, it was feasible for them to undertake this work, though they reported to be overburdened due to non-replacement of personnel whose job were shared with them. They were confident of using tablets since they had prior experience in using android tablets for the Government maternal and antenatal health services. There were no difficulties reported for using SMARThealth application. However, due to slow internet speed in the sample area, sometimes the data saving was problematic or delayed.

The SMARThealth application helped the ANMs to communicate about cardiovascular disease risk and educate the participants and family members about diet, tobacco use and physical activity. The graphical and colorful visual display on the tablet were also very useful for the ANMs to motivate the patients to adhere to medications.

“*We inform them the necessary precautions to keep their BP and sugar levels in control by regulating diet, walking regularly so that they remain healthy ……the ICDM application also has audio and video, this is helpful. It has 4-5 types of videos, we show them to the patients” – ANM 4*

ANMs were found to be already overburdened with the responsibilities of several community-based health services and disease control programs. In addition, staff attrition and non-replacement of positions, has led to extra burden on remaining workforce.

“*workload has increased drastically. for every 5000 population there must be two ANMs. currently I'm the one in this subcenter. My senior ANM got promotion and she left, so that post is vacant. I've to take responsibility of 5000 population alone.” – ANM 5*

Since the ICDM program aligned its visit with the DOTS schedule, the ANMs did not perceive this as inconvenient.

### Patient Experience

The intervention was acceptable to the study participants who considered the process helpful for them to receive services on a regular basis at their doorstep. They liked the concept of free screening, education, and support by the ANM at their home, without having to lose daily wages for hospital visits and spending money on medications in a private pharmacy.

“*If we go to private hospitals, they will give medicines which cost thousands…private doctors don't counsel us…ANMs do all tests, check their BP, sugar and give them medicines for free and make them sit for 15 min and counsel them..” Patient 3*

“*Without getting tired and without loss of money, they are coming to our place and conducting regular check-ups and monthly tests. We are not losing our wages.” – Patient 5*

Watching the video about risk factors and health messages on tablet and graphical presentation of their risk levels comparing from previous visits were motivating for them. They felt good to receive advice and reminders on medication use.

## Discussion

WHO's TB-DM collaborative framework 2011 emphasized that implementation of co-management strategies is crucial to curtail the rising TB-DM co-epidemic (1). The framework also recommended research and evaluation studies to be carried out to gather evidence on how local and national health programs and systems can be adapted to achieve effective co-management strategies (1). The study findings suggest feasibility of TB-DM co-management within the existing health care systems of India with sufficient training and properly planned implementation of intervention. This is consistent with findings of other similar studies in Mexico, Tanzania, and Nigeria (2–4). Several other studies have already established the possibility of bidirectional screening of TB-DM within the health programs of many countries including India (5–7).

An Ethiopian study reported good acceptability of the integrated TB-DM care within the TB control program (8). Task sharing approach, digital intervention and door-step free care delivery were found to be the main enablers during this TB-DM co-management intervention. Nevertheless, inadequate awareness, training and overburdened community health workers emerged as the key barriers.

Task sharing with the non-physician health workers have been proven to be feasible, safe, and cost-effective model in various contexts to combat growing burden of diseases (9–11). In this study, task sharing for combined management of TB-DM supported the earlier evidence of being feasible in a community care setting. Delivery of supportive care intervention for DM and other cardiovascular diseases at the patient's doorstep by the community health workers was found to be a key factor in achieving patient satisfaction for this intervention strategy.

Recent studies have demonstrated the effectiveness of computerized clinical decision support systems (CDSS) to screen individuals and provide individualized recommendations for appropriate healthcare delivery (12, 13). This study also reported the use of digital intervention with the SMARThealth application to be acceptable by the community health workers, patients, and their family members in elevating motivation level of patients for better adherence to medications and lifestyle modifications. By viewing the pictorial and graphical presentation of their health indicators during the routine visits of health workers was reported to be enticing for the patients and it kept their motivation up throughout the intervention period.

While there was minimal awareness among the non-physician health workers about TB-DM coexistence at the beginning of the study, interactive training helped in raising their knowledge about this comorbidity, their screening methods and healthier lifestyle behaviors. Community health workers play a pivotal role in most public health programs in India especially for the community level activities, awareness, and care delivery. Lately, they have become overburdened with many programs competing for their time for service implementation as well as extensive documentation of the activities. This study found such complains of work overburden by the community health workers. However, scheduling intervention visits on the days of routine DOTS visits in this study enabled the community health workers to manage both tasks without much inconvenience.

In this study, the prevalence of DM among the TB patients was observed to be 15.8%. This is consistent with a Madhya Pradesh study in 2017 which screened TB patients at DOTS centers and reported the DM prevalence to be 15.4% (14). Other two Indian studies have reported DM prevalence of 11.9 and 8%, respectively among the TB patients (15, 16).

This randomized pilot study was not effective in lowering blood glucose levels of patients in the intervention group. This may be attributed to smaller sample size of this pilot study trial which aimed to demonstrate feasibility over a short intervention period. The study demonstrated a non-significant decline in the prevalence of risky behaviors like smoking and alcohol consumption through the course of the intervention. A similarly unclear result was found for medication adherence in this study.

Additional evidence using large-scale randomized controlled trial design is needed to confirm feasibility of managing TB-DM comorbid patients within the robust TB control program. While the DOTS TB strategy is designed for 6–12 months depending on the severity of infection, management of chronic diseases like DM and cardiovascular diseases (CVDs) require long-term treatment approaches. There is need to institutionalize bi-directional screening of patients with TB and DM and screen individuals for other co-morbidities such as hypertension which would impact the outcomes. WHO's TB-DM collaborative framework and India's TB strategy recommends screening of TB and DM patients (25). The framework also encourages implementation research to contextualize to local health systems. There is a need to invest in robust evidence generation to develop a strong community based integrated care protocol with detailed guide on screening, referral, follow-up, and continued treatment for TB-DM comorbid patients. The presence of TB and DM represents the double burden of disease which needs to be addressed by health systems, especially in LMICs. Primary health systems need to be responsive, and services need to be adapted to address these comorbidities.

The population based non-communicable disease screening program in India currently requires a basic CVD risk assessment at the community level by the ASHAs followed by referral and opportunistic screening of BP, RBS and some of the common cancers of a targeted group of population above 30 years of age by the ANMs at the sub-center level (17). India's national framework for joint TB-DM collaborative activities 2017 mandates bidirectional screening and referral of TB and DM patients in India (26). However, it remains fully clear on the follow-up and joint intervention activities for comorbid patients. Subsequently, the Community Based Assessment Checklist (CBAC) form being used in India still remain standalone in terms of early risk assessment of common NCDs and TB with some referral instructions for TB suspected cases for further diagnosis to the TB care center (18).

While everyone referred to health facilities for further detection and care of the comorbidity may not visit there, the target of providing integrated care for TB-DM remains far from being full-proof. Also, the population based screening process is recommended to be followed for patients with DM once a year by the ANMs (17), whereas patients with TB-DM comorbidity need more frequent screening and follow-up considering their higher susceptibility toward severity and fatality. Hence, a detailed integrated intervention pathway on community-based follow-up and care for patients with TB-DM comorbidity in India is essential for preventing serious consequences of this deadly comorbidity and save lives.

## Data Availability Statement

The raw data supporting the conclusions of this article will be made available by the authors, without undue reservation.

## Ethics Statement

The studies involving human participants were reviewed and approved by an Independent Ethics Review Committee of the Centre for Chronic Disease Control, New Delhi, India. The patients/participants provided their written informed consent to participate in this study.

## Author Contributions

RJ conceived the idea for the study, its design, and led the writing of the manuscript. DB carried out the qualitative analysis and contributed to the writing of the manuscript. GD carried out the quantitative analysis and contributed to the writing of the manuscript. MA supervised the implementation of the study project. KY contributed to the writing of the manuscript. DP led the design of the study, its implementation, and contributed to the writing of the study. All authors contributed to the article and approved the submitted version.

## Funding

RJ was funded by an Australian National Heart Foundation Fellowship and UNSW Scientia Fellowship. KY was supported by a UNSW Scientia Scholarship.

## Conflict of Interest

The authors declare that the research was conducted in the absence of any commercial or financial relationships that could be construed as a potential conflict of interest.

## Publisher's Note

All claims expressed in this article are solely those of the authors and do not necessarily represent those of their affiliated organizations, or those of the publisher, the editors and the reviewers. Any product that may be evaluated in this article, or claim that may be made by its manufacturer, is not guaranteed or endorsed by the publisher.
